# RESPIRATORY MUSCLE IMPAIRMENT EVALUATED WITH MEP/MIP RATIO IN CHILDREN AND ADOLESCENTS WITH CHRONIC RESPIRATORY DISEASE

**DOI:** 10.1590/1984-0462/2021/39/2019414

**Published:** 2020-12-18

**Authors:** Iván Rodríguez-Núñez, Gerardo Torres, Soledad Luarte-Martinez, Carlos Manterola, Daniel Zenteno

**Affiliations:** a Universidad de Concepción, Chile.; b Dr. Guillermo Grant Benavente Hospital, Concepción, Chile.; c Universidad de La Frontera, Chile.

**Keywords:** Muscle strength, Respiratory muscles, Muscle fatigue, Muscle weakness, Neuromuscular disease, Bronchiolitis obliterans, Força muscular, Músculos respiratórios, Fadiga muscular, Fraqueza muscular, Doença neuromuscular, Bronquiolite obliterante

## Abstract

**Objective:**

To evaluate the strength of respiratory muscles and to compare maximum inspiratory (MIP) and expiratory (MEP) pressure and MEP/MIP ratio between patients with chronic respiratory diseases and healthy individuals.

**Methods:**

Case-control study. Individuals with neuromuscular disease and post-infectious bronchiolitis obliterans were considered. In addition, they were also matched according to anthropometric and demographic characteristics with healthy children and adolescents. MIP, MEP in the three groups, and pulmonary function only in patients with chronic respiratory diseases were recorded.

**Results:**

A total of 52 subjects with CRD (25 with neuromuscular disease, and 27 with post-infectious bronchiolitis obliterans) and 85 healthy individuals were included, with an average age of 11.3±2.1 years. Patients with neuromuscular disease and post-infectious bronchiolitis obliterans presented lower MIP and MEP when compared with healthy individuals, although MEP/MIP ratio was lower in patients with neuromuscular disease (0.87±0.3) and higher in patients with post-infectious bronchiolitis obliterans (1.1±0.3) compared to the healthy group (0.97±0.2). Only in patients with neuromuscular disease a negative correlation was observed between MEP/MIP ratio and age (r=-0.50; p=0.01).

**Conclusions:**

Differences in the pattern of muscular weakness between patients with chronic respiratory diseases were observed. In patients with neuromuscular disease, a decrease in the MEP/MIP ratio depending on MIP was verified; and in those patients with post-infectious bronchiolitis obliterans, an increase in the MEP/MIP ratio depending on MIP was also observed.

## RESUMO

**Objetivo:**

Avaliar a força dos músculos respiratórios e comparar a relação entre a pressão expiratória máxima (PEmáx) e a pressão inspiratória máxima (PImáx) em pacientes com doença respiratória crônica (DRC) e crianças saudáveis.

**Métodos:**

Estudo caso-controle. Foram selecionados indivíduos com doença neuromuscular e bronquiolite obliterante pós-infecciosa. Ademais, os grupos foram pareados com crianças e adolescentes saudáveis, considerando características antropométricas e demográficas. Foram registradas a PImáx e a PEmáx nos três grupos e a função pulmonar apenas em pacientes com doença respiratória crônica.

**Resultados:**

Foram incluídos 52 indivíduos com DRC (25 com doença neuromuscular e 27 com bronquiolite obliterante pós-infecciosa) e 85 indivíduos saudáveis, com idade média de 11,3±2,1 anos. Pacientes com doença neuromuscular e bronquiolite obliterante pós-infecciosa apresentaram menor PImáx e PEmáx em comparação aos indivíduos saudáveis, embora a relação PEmáx/PImáx tenha sido menor nos pacientes com doença neuromuscular (0,87±0,3) e maior nos pacientes com bronquiolite obliterante pós-infecciosa (1,1±0,3) em comparação ao grupo saudável (0,97±0,2). Somente em pacientes com doença neuromuscular foi observada uma correlação negativa entre a razão PEmáx/PImáx e a idade (r=-0,50; p=0,01).

**Conclusões:**

Foram observadas diferenças no padrão de fraqueza muscular em pacientes com doença respiratória crônica. Nos pacientes com doença neuromuscular, verificou-se diminuição na relação PEmáx/PImáx dependendo da PImáx; em pacientes com bronquiolite obliterante pós-infecciosa, foi observado aumento na relação dependendo da PImáx.

## INTRODUCTION

The functional deterioration of respiratory muscles is considered a frequent complication in chronic respiratory diseases (CRD).^1^ ^,^ ^2^ ^,^ ^3^ Several studies have revealed the existence of a significant drop in strength and resistance of inspiratory and expiratory muscles in children and adolescents with chronic pulmonary damage and neuromuscular diseases (NMD),^4^ ^,^ ^5^ ^,^ ^6^ ^,^ ^7^ which promotes the development of hypoventilation, microatelectasis, and deterioration of cough mechanisms, altering their prognosis.^4^ ^,^ ^8^ ^,^ ^9^

One of the most prevalent CRD in our pediatric population is the post-infectious bronchiolitis obliterans (PIBO), which occurs secondarily to a pulmonary infection during childhood.^10^ ^,^ ^11^ Among its complications, a severe functional deterioration secondary to pulmonary damage is highlighted.^12^ ^,^ ^13^ However, the magnitude of respiratory muscular deterioration has not been explored in this group of patients.

In the clinical context, the evaluation of the strength of respiratory muscles is performed with measurements of maximum inspiratory pressure (MIP) and maximum expiratory pressure (MEP) exerted through the mouth.^14^ ^,^ ^15^ This evaluation method has been validated in adults and children, and it has been used both for evaluating and monitoring patients with CRD.^16^ ^,^ ^17^ ^,^ ^18^ For this purpose, the absolute value of MIP and MEP expressed in cmH_2_O is considered, as well as its percentage value relative to a standard value calculated from reference equations, in which age, gender and anthropomorphic characteristics of the target population are often considered as predictive variables.^15^ ^,^ ^19^

Despite the above, independent values of MIP and MEP are not useful to analyze the imbalance between the magnitude of inspiratory and expiratory strength in a specific individual. Consequently, it has recently been shown that ­MEP/­MIP ratio could be a suitable and straight forward parameter for establishing with certainty the strength loss of respiratory muscles in healthy adults with paralysis of the phrenic nerve and progressive NMD.^20^ ^,^ ^21^

Until now, respiratory muscle balance in healthy children is unknown, as well as the pattern of muscular weakness in CRDs patients. Additionally, there is no evidence of respiratory muscle deterioration and variation in the MEP/MIP ratio in patients with PIBO. Therefore, the objective of the present study was to evaluate the strength of inspiratory and expiratory muscles in healthy children and adolescents, affected by NMD and PIBO, as well as to calculate MEP/MIP ratio and compare the value obtained between the study groups. We hypothesize that both children with NMD and PIBO have lower respiratory muscle strength when compared to healthy subjects, and present significant differences in the pattern of muscular weakness measured with the MEP/MIP ratio.

## METHOD

In this case-control study, the records of MIP, MEP, and pulmonary function of children and adolescents admitted in the Infant Pulmonary Rehabilitation Program of Dr. Guillermo Grant Benavente Hospital of Concepción, Chile, between 2011 and 2016, were selected. The existence of cognitive deficit and the presence of an acute clinical disorder that would have altered the basal state of patients within four weeks before the pulmonary function tests were considered as exclusion criteria.

Besides that, healthy individuals belonging to four public schools in Concepción City were invited to participate in the study. Members of this group were matched by age (±1 year old), gender (same gender), weight, and height (±5%) with patients with NMD and PIBO. The existence of obesity, cardiorespiratory or chronic neuromuscular disease, and the presence of any acute pathology during the four weeks before measuring variables were considered as exclusion criteria. Eligibility criteria were verified with a checklist completed by parents.

Parents or legal tutors of each participant in the study signed an informed consent form, and children younger than 12 signed an informed assent. The study was approved by the Scientific Ethical Committee of Dr. Guillermo Grant Benavente Hospital of Concepción.

Sample size was estimated considering a 5% of type I risk error, the statistical power of 99%, a standard deviation of 20 cm H_2_O, and a minimum clinically significant difference in the MIP of 20 cm H_2_O between healthy individuals and those affected with CRD. Moreover, a 1:3 ratio between patients with CRD and healthy individuals was considered. Thus, a minimum of 25 individuals in NMD and PIBO groups, as well as 75 individuals in the control group were estimated to perform the study.

In the three groups under study, variables such as age, gender, height, weight, MIP, and MEP were recorded. Both weight and height were determined by an analogous scale and a metric tape. On the other hand, pulmonary function parameters were only recorded from patients with NMD and PIBO.

Pulmonary function was determined by spirometry, which was evaluated according to the protocol established by the European Respiratory Society and the American Thoracic Society ­(ERS/­ATS).^22^ The parameters considered in the study were forced expiratory volume at first second (FEV_1_), forced vital capacity (FVC), FEV_1_/FVC index, and forced expiratory flow between 25% and 75% of FVC (FEF_25-75_.). Results were expressed in absolute values and percentages of predictive value, according to Knudson et al.^23^ Pulmonary function was determined with a Microlab ML3500 spirometer (Micro Medical Ltd, Rochester, England).

The strength of respiratory muscles was determined by the maximum inspiratory pressure (MIP), measured with a maximum inspiratory effort maintained for at least one second from the residual volume. On the other hand, the maximum expiratory pressure (MEP) was measured with the maximum respiratory effort maintained for at least one second from the total pulmonary capacity.^14^ The best MIP and MEP values from three acceptable and reproducible attempts was recorded. Values obtained were expressed in absolute values (cm H_2_O) and percentages according to Chilean predictive values published by Contreras et al.^24^

The tools used to measure muscle strength were a digital pressure meter (MicroRPM; Vyaire Medical Inc., Mettawa, IL, USA) and an aneroid pressure gauge (Vacuum/Pressure Gauge NS120-TRS; Instrumentation Industries, Inc. Bethel Park, USA) calibrated in centimeters of water (0 to -120 and 0 to +120 cm H_2_O). Equipment showed to be valid and reliable to determine the strength of inspiratory and expiratory muscles.^24^ ^,^ ^25^

Exploratory analysis of data was performed with normality evaluation using the Shapiro Wilk test. Subsequently, descriptive statistic was performed with the calculation of average and standard deviation for quantitative variables, and percentage for qualitative variables. Chi-square was used to evaluate the difference of proportions of qualitative variables, Student’s t-test for independent samples to compare parameters of pulmonary function between groups of patients with CRD, and Student’s t-test for related samples to compare MEP/MIP ratio with the %MEP/%MIP, for each study group. On the other hand, Levene’s test was used to evaluate heterogeneity of variances. Therefore, in order to compare quantitative variables between the three groups under study when there was no statistical significance in heterogeneity of variances, one-way ANOVA and Scheffé *post hoc* test were used. When there was heterogeneity of variances, the Kruskal Wallis test was used. Finally, Pearson’s correlation coefficient was used to determine the correlation between the MEP/MIP ratio (and %MEP/%MIP ratio) with both parameters of respiratory muscle strength (MIP and MEP) and with the age of participants. The analysis was performed by using the statistic software MedCalc, version 17.4 (MedCalc Software bvba, Ostend, Belgium), and a value of p<0.05 was considered as statistically significant.

## RESULTS

**Figure 1:**
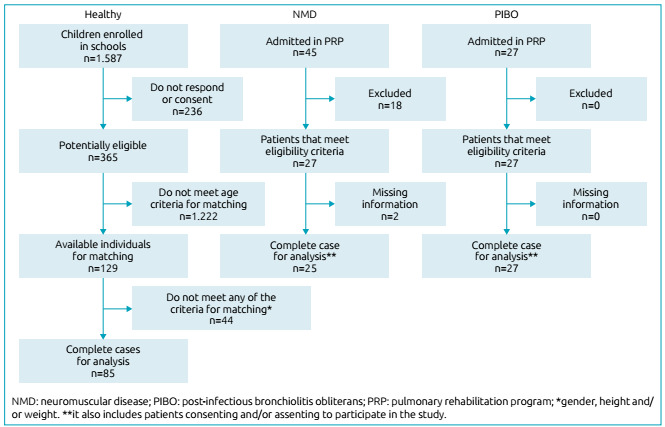
Flowchart of recruitment of study subjects.

The study included 52 individuals with CRD and 85 healthy individuals. Of the patients with CRD, 25 presented NMD, and 27, PIBO. Flowchart of recruitment of study subjects is shown in Figure 1. No significant differences were observed between groups as to age, gender, and anthropometric variables (Table 1).

**Table 1:** General characteristics of the study groups.

	Healthy (n=85)	NMD (n=25)	PIBO (n=27)	p-value*
Age (years old)	11.1±1.6	11.4 ± 2.9	11.7±2.8	0.384*
Gender (M/F)	47/38	18/7	19/8	0.179**
Weight (kg)	47.5± 13.1	46.5±13.2	44.1±11.4	0.483*
Height (cm)	149.4± 10.5	145.5±9.5	151.3±11.4	0.300*

NMD: neuromuscular disease; PIBO: post-infectious bronchiolitis obliterans; M: male; F: female. *One-way ANOVA; **chi-square test was used.

The group of patients with NMD consisted of patients with Duchenne muscular dystrophy (n=13; 52.0%), congenital myopathy (n=4; 16.0%), Type II spinal atrophy (n=3; 12.0%), Becker muscular dystrophy (n=1; 4.0%), fascioscapulohumeral dystrophy (n=1; 4.0%), and Bethlem myopathy (n=1; 4.0%).

Regarding pulmonary function, FEV_1_ (%), FEF_25-75_ (absolute value and percentage) and FEV1/FVC ratio were lower in patients with BIPO, compared to patients with NMD (p<0.05). Additionally, the existence of a restrictive ventilatory pattern was observed in children with NMD, and an obstructive ventilatory pattern, in children with PIBO (Table 2).

**Table 2:** Spirometric values of patients with chronic respiratory disease.

	NMD (n=25)	PIBO (n=27)	p-value*
FEV_1_ (l)	1.6±0.5	1.5±0.6	0.338
FEV_1_ (%)	82.0±24.2	65.5±20.1	0.011
FVC(l)	1.9±0.6	2.3±0.9	0.080
FVC (%)	79.1±28.8	88.2±19.6	0.071
FEV_1_/ FVC	87.6±7.7	64.9±13.8	<0.001
FEF_25-7 5_ (l/m)	2.0±1.2	1.0±0.7	<0.001
FEF_25-75_ (%)	76.6±30.7	37.3±22.0	<0.001

NMD: neuromuscular disease; PIBO: post-infectious bronchiolitis obliterans; FEV1: forced expiratory volume during the first second; FVC: forced vital capacity; FEF25-75: forced expiratory flow between the 25 and 75 percent of the forced vital capacity. Results are shown in absolute values (mean and standard deviation) and predictive values according to Knudson et al. *Independent sample Student’s t-test was used.

Results of respiratory muscle strength and MEP/MIP ratio are shown in Table 3. Both patients with NMD and PIBO presented MIP and MEP values lower than those of healthy individuals. On the other hand, patients with NMD presented lower absolute values in MIP, MEP, and %MEP compared to patients with PIBO. Additionally, a positive correlation was observed between MIP and MEP in healthy (r=0.60; p<0.001), NMD (r=0.58; p=0.021), and PIBO (r=0.60; p=0.001).

**Table 3:** Respiratory muscle strength and the maximal expiratory pressure/maximal inspiratory pressure ratio of the study groups.

	Healthy (n=85)	NMD (n=25)	PIBO (n=27)	p-value*
MIP (cmH_2_O)	103.4±16.4	56.8±19.3^a.b^	68.4±24.8^a^	<0.001
MIP (%)	100.8±16.6	54.9±18.1^a^	65.6±24.8^a^	<0.001
MEP (cmH_2_O)	100.2±21.7	47.2±18.3^a.b^	72.5±21.2^a^	<0.001
MEP (%)	75.2±17.0	34.9±15.4 ^a.b^	52.6±19.2^a^	<0.001
MEP/MIP ratio	0.97±0.1	0.87±0.3 ^a.b^	1.11±0.4	0.017*
%MEP/%MIP ratio	0.75±0.1	0.64±0.2 ^a.b^	0.85±0.3	0.004*

NMD: neuromuscular disease; PIBO: post-infectious bronchiolitis obliterans; MIP: maximal inspiratory pressure; MEP: maximal expiratory pressure; %: percentage of predictive values according to Contreras et al. ^a^: statistical difference with healthy group (p<0.05); ^b^: statistical difference with PIBO group (p<0.05). *Kruskal-Wallis test.

Regarding the MEP/MIP ratio, there was a significant difference between the absolute and relative values in the three groups (p<0.001). In addition to that, statistically significant differences were observed between groups in both MEP/MIP ratio and in the %MEP/%MIP ratio. *Post-hoc* analysis indicated that the group of patients with NMD presented a lower MEP/MIP ratio and %MEP/%MIP ratio compared to patients with PIBO and healthy individuals (Table 3).

On the other hand, in both NMD and PIBO patients, respiratory muscle function was correlated with MEP/MIP ratio; however, in healthy individuals, only MEP was correlated with MEP/MIP ratio. Moreover, in healthy individuals and patients with NMD, only %MEP was correlated with %MEP/%MIP ratio and, in patients with PIBO, only %MIP was correlated with %MEP/%MIP (Table 4).

**Table 4:** Correlation between the parameters of respiratory muscle strength and maximal expiratory pressure/maximal inspiratory pressure ratio.

	Healthy (n=85)	NMD (n=25)	PIBO (n=27)
MIP (cmH_2_O)	-0.17	-0.44*	-0.40*
MIP (%)^a^	-0.10	-0.24	-0.46*
MEP (cmH_2_O)	0.67***	0.42*	0.43*
MEP (%)^a^	0.69***	0.60**	0.32

NMD: neuromuscular disease; PIBO: post-infectious bronchiolitis obliterans; MIP: maximal inspiratory pressure; MEP: maximal expiratory pressure; %: percentage of predictive values according to Contreras et al.; ^a^: MIP and MEP expressed as relative value were correlated with %MEP/%MIP ratio; *<0.05; **<0.01; ***<0.001.

**Figure 2:**
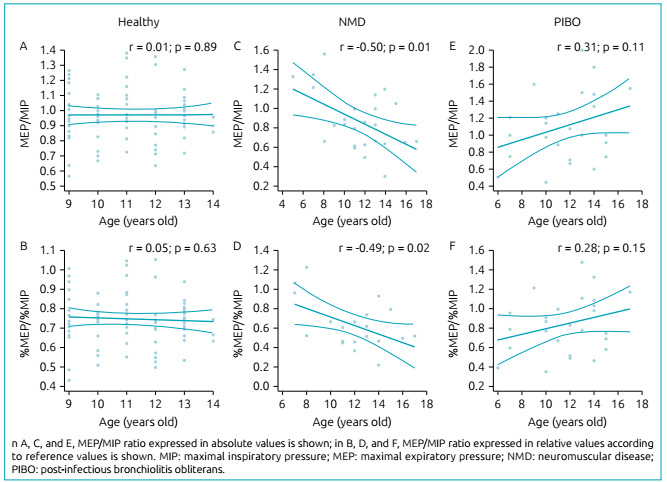
Correlation between maximal expiratory pressure/maximal inspiratory pressure ratio and age.

Correlation between MEP/MIP ratio and the age of individuals at the time of evaluation is presented in Figure 2. A negative correlation was observed between MEP/MIP and %MEP/%MIP ratios with age only in patients with NMD (Figures 2 C and D).

## DISCUSSION

The measurement of the respiratory muscles is a frequent practice in the evaluation and follow-up of patients with NMD and PIBO.^15^ ^,^ ^26^ In this context, the present study aimed to analyze the pattern of respiratory muscles in children and adolescents with NMD and PIBO with the MIP/MEP ratio. Consequently, both weakness of respiratory muscles in both groups and significant differences in the pattern of inspiratory/expiratory muscular deterioration were observed. Thus, given that patients with NMD experienced substantial drops in the MEP/MIP ratio, patients with PIBO presented an increase in the magnitude of this variable.

These results are in accordance with those reported by Fregonezi et al., who observed significant differences in ­MEP/­MIP index in patients with different types of neuromuscular diseases. In their study, a lower MEP/MIP ratio was observed in patients with myotonic dystrophy, which represents a more significant muscular weakness in this group of patients.^21^

In our study, the patients with NMD presented a 10.0% lower MEP/MIP ratio when compared to healthy individuals. Furthermore, most patients with NMD (52.0%) had Duchenne Muscular Dystrophy (DMD). In this sense, several studies have revealed the existence of weakness in respiratory muscles in patients with different types of muscular dystrophy, which is associated to a significant deterioration of coughing capacity.^27^ ^,^ ^28^ This was confirmed by our results, in which the ­MEP/­MIP ratio was mainly associated to the strength of expiratory muscles given the existence of a significant correlation between %MEP/%MIP with %MEP, but not with the %MIP (Table 4).

Moreover, the MEP/MIP ratio was inversely correlated with age only in patients with NMD. Previous studies agree with our findings by reporting the existence of a pattern of progressive respiratory muscle deterioration (mainly affecting the expiratory muscles) in patients with progressive NMD.^4^ ^,^ ^29^ ^,^ ^30^

Concerning patients with PIBO, several studies have revealed the existence of a secondary functional deterioration to pulmonary damage and a lower physical capacity.^12^ ^,^ ^13^ ^,^ ^31^ However, to date, no reports are showing respiratory muscle impairment in these patients. In our study, patients with PIBO experienced a 33.8% lower level of inspiratory muscle strength, and 26.7% of all patients, a lower level of expiratory muscle strength when compared to the group with healthy individuals. The magnitude of deterioration is similar to that observed in other chronic pulmonary diseases.^6^ ^,^ ^32^ ^,^ ^33^

In addition, patients with PIBO experienced a higher ­MEP/­MIP ratio compared to patients with NMD (p<0.05). Additionally, the variations in MEP/MIP ratio was mostly due to changes in the magnitude of the strength of inspiratory muscles. These results are in accordance with those from studies carried out in patients with other chronic pulmonary diseases,^7^ ^,^ ^17^ ^,^ ^33^ ^,^ ^34^ ^,^ ^35^ which have revealed the existence of weakness of inspiratory muscles associated to the induction of mechanisms of oxidative stress, apoptosis, and inspiratory muscular atrophy.^1^ ^,^ ^2^ ^,^ ^36^

Although there are no data regarding potential mechanisms underlying the functional deterioration observed in patients with PIBO, some studies observe a high level of oxidative stress in lung parenchyma,^37^ ^,^ ^38^ ^,^ ^39^ suggesting the possibility that inspiratory muscle weakness seen in these patients may be associated to oxidative mechanisms similar to those observed in patients with chronic obstructive pulmonary disease (COPD).^1^

To the best of our knowledge, the present study constitutes the first report that indicates the magnitude of respiratory muscle weakness in patients with PIBO, as well as the differences in the MEP/MIP ratio in children and adolescents with CRD when compared to healthy individuals. In this context, the recognition of a variable pattern of respiratory muscle weakness in patients with CRD has direct implications on rehabilitation, because such data allow focusing respiratory muscle training protocols on the muscle group that will be primarily affected according to the specific pattern of muscle deterioration of each patient. Despite this, determining whether muscle training focused on respiratory muscles is effective in recovering inspiratory/expiratory balance is needed, which should be explored in further research.

The present study has certain limitations to be discussed. Among them, the verification of exclusion criteria in the group of healthy individuals is highlight, which was performed with a checklist that was completed by parents, and no pulmonary function tests were conducted to identify the existence of any respiratory pathology in this group. Additionally, the %MEP was less than the normal value (75.2%). Therefore, it is not possible to rule out that the control group may be underrepresenting the healthy population, which could result in the presence of an eventual sampling bias.

On the other hand, patients with NMD presented different diagnoses, which impede to conclude for a specific type of NMD. Consequently, the existence of individual differences regarding the pattern of respiratory muscle weakness between different types of NMD could be feasible.

Finally, children and adolescents with NMD and PIBO presented weaknesses of respiratory muscle and experienced differences in the pattern of respiratory muscular weakness. Patients with NMD had a decrease in the MEP/MIP ratio associated with a lower strength of expiratory muscles and higher age of subjects, whereas in patients with PIBO there was an increase in the MEP/MIP ratio associated with a lower strength of inspiratory muscles.
